# Enhanced antitumor effects of low-frequency ultrasound and microbubbles in combination with simvastatin by downregulating caveolin-1 in prostatic DU145 cells

**DOI:** 10.3892/ol.2014.2005

**Published:** 2014-03-28

**Authors:** WEI-PING XU, E. SHEN, WEN-KUN BAI, YU WANG, BING HU

**Affiliations:** 1 Department of Ultrasound in Medicine, Shanghai Jiao Tong University Affiliated Sixth People’s Hospital, Shanghai Institute of Ultrasound in Medicine, Shanghai 200233, P.R. China; 2 Department of Ultrasound, Shanghai Minhang District Central Hospital of Ruijin Hospital Group, Shanghai 201199, P.R. China

**Keywords:** low frequency ultrasound and microbubbles, simvastatin, apoptosis, caveolin-1, prostate cancer

## Abstract

Advanced prostate cancer is difficult to treat due to androgen resistance, its deep location and blood tumor barriers. Low-frequency ultrasound (LFU) has potential clinical applications in the treatment of prostate cancer due to its strong penetrability and high sensitivity towards tumor cells. Simvastatin has often been administered as a preventive agent in prostate tumors. The aim of the present study was to investigate the enhanced effects of LFU and microbubbles in combination with simvastatin, in inhibiting cell viability and promoting apoptosis of androgen-independent prostatic DU145 cells. Cultured DU145 cells were divided into six groups based on the combination of treatments as follows: Control, LFU, LFU and microbubbles (LFUM), simvastatin, LFU and simvastatin, LFUM and simvastatin. The cells were treated by LFU (80 kHz) continuously for 30 sec with an ultrasound intensity of 0.45 W/cm^2^ and a microbubble density of 20%. Simvastatin was added 30 h prior to the ultrasound exposure. The results indicated that cell viability was marginally reduced in the LFU and simvastatin alone treatment groups compared with the control 24 h following ultrasound exposure. The combination of LFU, with microbubbles or simvastatin, potentiated the growth inhibition; the greatest inhibition was observed in the cells that were subject to treatment with LFUM and simvastatin in combination. Furthermore, this inhibitory effect was enhanced in a time-dependent manner. For cell apoptosis, a low dose of simvastatin had no apparent affect on the DU145 cells, while LFU marginally promoted cell apoptosis. Microbubbles or simvastatin increased the apoptosis rate of the DU145 cells, however, the combination of LFUM and simvastatin induced a strong synergistic effect on cell apoptosis. Western blotting analysis demonstrated a high expression level of caveolin-1 in resting DU145 cells. LFUM or combined LFU and simvastatin resulted in a greater reduction in the expression compared with the control group (P<0.05). The expression of caveolin-1 was lowest in the LFUM combined with simvastatin treatment group. The expression of phospho-Akt (p-Akt) was consistent with caveolin-1, with the lowest expression levels of p-Akt observed in the cells that were treated with the combination of LFUM and simvastatin. The results indicate that LFUM in combination with simvastatin may additively or synergistically inhibit cell viability and induce apoptosis of DU145 cells by downregulating caveolin-1 and p-Akt protein expression.

## Introduction

Prostate cancer is the second leading cause of cancer-associated mortality in males in the USA (1). The incidence of prostate cancer in China, which is gradually becoming an aging society, has been increasing by 9.2% each year from 2001 to 2010 according to statistics released by the Beijing Bureau of Health (2). The majority of cases are initially androgen-dependent, which respond well to androgen ablation therapy and radical prostatectomy, however, ~30% of patients progress to androgen-independent prostate cancer (AIPC) or to hormone-refractory prostate cancer (HRPC) (3). Currently, there are no therapeutic options that will effectively cure patients with AIPC or HRPC. A number of studies have demonstrated that caveolin-1 is overexpressed and that its upregulation is positively correlated with cell proliferation and progression in AIPC or HRPC (4,5). Caveolin-1 is, therefore, a biomarker and therapeutic target for prostate carcinomas.

Simvastatin, a commonly prescribed medication for treatment of hypercholesterolemia, is a class of inhibitors of hydroxylmethylglutaryl-coenzyme A reductase, the rate-limiting enzyme in the mevalonate pathway. Previous studies demonstrated that simvastatin reduced the risk of total and clinically advanced prostate cancer (6,7). However, long-term simvastatin use was associated with a significant increase in prostate cancer risk and other side-effects (8). Furthermore, it was demonstrated that high-dose simvastatin had cytostatic effects on normal cells (9). Therefore, lowering the toxic effects of simvastatin is urgently required, to facilitate its clinical utility as an anticancer agent.

Diagnostic medical ultrasound has extensive uses in clinical practice and is increasing in its therapeutic applications. Advantages of ultrasound therapy are that it is non-invasive, safe and inexpensive. Previous studies have demonstrated that cancer cells are more susceptible than normal cells to ultrasound (10,11), which has served as the experimental foundation for its use as a cancer treatment. It was discovered that low-frequency ultrasound (LFU) has a sonoporative effect by increasing the permeability of the cell membrane to facilitate the transport of macromolecules into the cell (12). When combined with microbubbles, LFU has improved its antitumor function via a cavitation effect (13). However, this antitumor effect is limited and the underlying mechanism is unclear. In the present study, the effects of LFU and microbubbles (LFUM) in combination with low-dose simvastatin on cell viability and apoptosis of DU145 cells were investigated and the possible mechanisms underlying this effect were examined.

## Materials and methods

### Ethical approval

The present study obtained permission from the ethics committee of the Shanghai Jiao Tong University Affiliated Sixth People’s Hospital and the Shanghai Institute of Ultrasound in Medicine (Shanghai, China).

### Cell culture

DU145 human prostate cancer cell line was purchased from the cell bank of the Chinese Academy of Sciences (Shanghai, China). The cells were cultured in RPMI-1640 medium supplemented with 10% fetal bovine serum in a humidified atmosphere of 5% CO_2_ at 37°C. The cultures that were at 70–80% confluence were used. The cells were divided into six groups according to their treatment combinations: Control, LFU, LFUM, simvastatin (Sigma, St. Louis, MO, USA), LFU and simvastatin, LFUM and simvastatin. The control cells were treated with sham irradiation. Simvastatin was added with a final concentration of 3 μM at 30 h prior to ultrasound exposure. This dose of simvastatin (3 μM) was considered to be non-toxic, as this level resulted in <5% cell death by trypan blue staining (data not shown).

### Microbubbles and ultrasonic irradiation

The ultrasound contrast agent Sonovue (Bracco, Milan, Italy) was reconstituted in 5 ml saline solution according to the manufacturer’s instructions, resulting in a preparation containing 2–5×10^8^ microbubbles/ml.

The low-frequency ultrasonic processor consisted of the ultrasonic generator, the single-channel amplifier and a flat transducer, developed by the Shanghai Institute of Ultrasound in Medicine (Shangai, China). The emission frequency was 80 kHz with tunable power between 0 and 3 W. The circular plate (diameter, 13 mm) at the front end of the transducer was mounted with stainless steel stents with the irradiated surface facing upwards. The concentration of DU145 cells was adjusted to 10^6^ cells/ml following a treatment of trypsin digestion. The cell suspension was aliquoted into a 1.5-ml eppendorf tube (diameter, 13 mm), which was placed upside down on the surface of the ultrasound probe and connected by the sonographic gel.

In the initial part of the present study, the orthogonal experimental design method was performed to identify the optimal experimental conditions for inducing prostate cancer cell apoptosis. According to influencing factors and previous knowledge, three factors were selected and each factor was divided into three levels as follows: Ultrasound intensity, 0.15, 0.30 and 0.45 W/cm^2^; irradiation time, 10, 20 and 30 sec; and microbubbles/cell suspension volume ratio, 10, 20 and 50%. The ultrasonic irradiation was subsequently conducted using the orthogonal design table with the three factors at the three levels. The cells continued to culture for 24 h following treatment and cell apoptosis was detected by flow cytometry (FACSAria II; BD Biosciences, Franklin Lakes, NJ, USA).

### 3-(4,5-dimethylthiazol-2-yl)-2, 5-diphenyltetrazolium bromide (MTT) assay

Cell viability was measured by the MTT assay (Wellscan MK3; Ani Labsystems, Ltd. OY, Vantaa, Finland). Briefly, DU145 cells in the presence or absence of simvastatin were seeded at a density of 2×10^4^ cells/ml in 200 μl/well culture medium in a 96-well microtiter plate (Costar 3599; Corning, New York, NY, USA), followed by ultrasound and microbubble treatment. Following 24 and 72 h, 50 μl MTT reagent was added to the cell culture plate and incubated for 4 h at 37°C according to the manufacturer’s instructions. The MTT reagent was removed, and 150 μl dimethylsulfoxide was added to each well. The plates were agitated (THZ-C; Taicang Experiment Equipment Factory, Taicang, China) for 15 min to completely dissolve the crystals and absorbance was measured at 492 nm using an enzyme-linked immunosorbent assay plate reader from MTX Lab Systems, Inc. (Vienna, VA, USA). The percentage of viable cells was calculated as follows: Viability (%) = absorbance of the experimental group/absorbance of the control group × 100. Each experiment was performed in triplicate.

### Flow cytometry apoptosis detection

The DU145 cells were grown on six-well culture plates for 24 h following treatment. Cells were trypsinized and suspended in cold phosphate-buffered saline and the cell density was adjusted to 2×10^6^/ml. Following centrifugation (Biofuge Stratas; Kendro Laboratory Products GmbH, Langenselbold, Germany) at 2,162 × g for 10 min at 4°C, the supernatant was removed and resuspended in 200 μl binding buffer. The cells were incubated with 10 μl annexin V-fluorescein isothiocyanate and 5 μl propidium iodide, mixed gently and protected from light exposure for 15 min at room temperature prior to the flow cytometry.

### Western blot analysis

Western blot analysis was conducted to determine the expression levels of caveolin-1 and phospho-Akt (p-Akt). The DU145 cells were distributed into 6-well plastic plates following treatment and after a 24-h incubation, the cells were lysed using the MBST lysis buffer (25 mM MBS, pH 6.5, 0.15 mM NaCl_2_, 1% Triton X-100), and the precipitate was removed by centrifugation at 8,648 × g. A bicinchoninic acid reagent (Sigma) was used to determine the protein concentration. The proteins were separated by 10% SDS-polyacrylamide gel electrophoresis. The gel was transferred onto a polyvinylidene difluoride membrane, and stained to examine the transfer and locate the molecular weight markers. The membrane was sealed for 1 h using the Tris-buffered saline and Tween 20 (TBST) buffer (20 mM Tris base pH 7.6, 50 mM NaCl, 0.1% Tween-20) containing 5% non-fat dried milk. The goat anti-rabbit caveolin-1 primary antibody (1:1,000; Epitomics, Burlingame, CA, USA), was added and incubated with the membrane for 1 h, washed three times with TBST, incubated with horseradish peroxidase-conjugated secondary antibodies (1:2,000) for 1 h at room temperature and washed three times with TBST. The labeled proteins were visualized using an X-ray western blotting detection kit (Pierce ECL western blotting substrate, Thermo Scientific Pierce, Rockford, IL, USA). The same method was used for detecting p-Akt (Cell Signaling Technology, Inc., USA) and β-actin served as a control. The bands were scanned and processed by Photoshop CS6 (Adobe Inc., San Jose, CA, USA). The following formula was used for calculating the relative content of the protein: Relative protein content = (mean intensity of a band area - mean intensity of the background)/(mean intensity of the control band - mean intensity of the background). The resulting values were averaged for the three experiments and the standard deviations (SDs) were calculated.

### Statistical analysis

All data represented the mean value of at least three independent experiments. The results were expressed as the mean ± SD. Statistical significance was determined by one-way analysis of variance followed by the Student-Newman-Keuls method for multiple comparisons between the pairs with P<0.05. P<0.05 was considered to indicate a statistically significant difference.

## Results

### Orthogonal test to optimize ultrasound parameters

**Figure 1:**
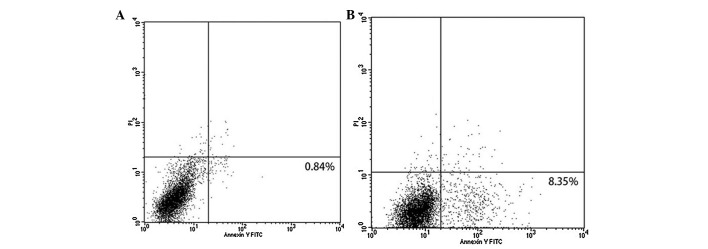
Proportion of DU145 cell apoptosis under the optimal experimental parameters. (A) Control (sham irradiation); (B) ultrasound intensity (0.45 W/cm^2^), irradiation time (30 sec), volume ratio of microbubbles to cell suspension (20%). PI, propidium iodide; FITC, fluorescein isothiocyanate.

In the pilot study, the optimal experimental parameters of DU145 cell apoptosis were determined using the orthogonal tests of three factors, each at three levels (Table I). Range analysis indicated that the R-values for the three parameters were ordered as follows: Ultrasound intensity > irradiation time > volume ratio of microbubbles to cell suspension; with ultrasound intensity exhibiting the greatest effect. The effect was correlated with the strength of the ultrasound intensity (0.45>0.30>0.15 W/cm^2^) or the duration of irradiation (30>20>10 sec), however, not for the ratio of microbubbles versus cell suspension volume (20>50>10%). Under the optimal combination of the three parameters (ultrasound intensity, 0.45 W/cm^2^; irradiation time, 30 sec; volume ratio of microbubbles to cell suspension, 20%), the rate of DU145 cell apoptosis was 8.35% (Fig. 1).

### Growth inhibition of DU145 cells by LFUM combined with simvastatin

**Figure 2:**
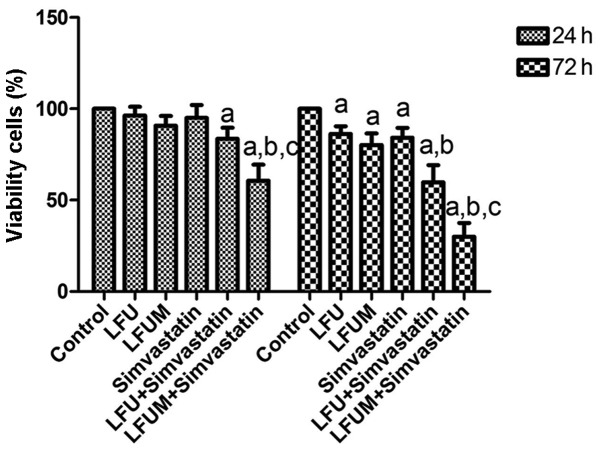
Effects of LFUM in combination with simvastatin on DU145 cell viability. The DU145 cells were treated with the indicated LFU, microbubbles and simvastatin for 24 and 72 h, and cells were subjected to the MTT assay as described in Materials and methods. Error bars represent the mean ± standard deviation of three independent experiments. ^a^P<0.05 compared with the control; ^b^P<0.05 compared with the LFUM-treated cells; ^c^P<0.05 compared with the combined LFU and simvastatin-treated cells. LFU, low-frequency ultrasound; LFUM, low-frequency ultrasound and microbubbles.

The MTT assay was used to evaluate cell viability and growth inhibition following treatment with LFU, microbubbles and simvastatin. The results demonstrated that cell viability was marginally reduced in the LFU (96.3±4.7%) or the simvastatin (95.1±7.1%) group compared with the control (100%) at 24 h following ultrasound exposure. The percentage of viable cells was 90.8±5.2, 83.6±6.1 and 60.6±8.8% in the LFUM group, LFU and simvastatin group, and LFUM and simvastatin group, respectively. When the time was prolonged to 72 h, the percentage of viable cells in the LFU, LFUM, simvastatin, LFU and simvastatin, and LFUM and simvastatin treatment groups was 86.4±4.1, 80.2±6.4, 84.1±5.4, 59.9±9.3 and 30.1±7.5%, respectively. Thus, growth inhibition of DU145 cells was enhanced in a time-dependent manner. These results indicated that the LFUM or LFU and simvastatin-treated cells exhibited improved growth inhibition compared with LFU or simvastatin alone. When used in combination, LFUM and simvastatin markedly increased the inhibitory effect of LFUM or LFU and simvastatin on DU145 cells. The difference was statistically significant (P<0.05; Fig. 2).

### Effect of LFUM combined with simvastatin on cell apoptosis

**Figure 3:**
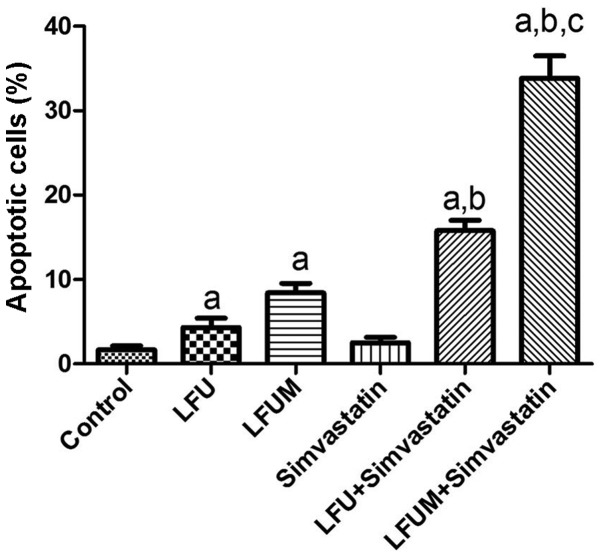
Effects of LFUM in combination with simvastatin on DU145 cell apoptosis. The DU145 cells were treated with the indicated LFU, microbubbles and simvastatin for 24 h and then subjected to flow cytometry. Error bars represent the mean ± standard deviation of three independent experiments. ^a^P<0.05 compared with the control; ^b^P<0.05 compared with the LFUM-treated cells; ^c^P<0.05 compared with the combined LFU and simvastatin-treated cells. LFU, low frequency ultrasound; LFUM, low frequency ultrasound and microbubbles.

Cell apoptosis was assessed at 24 h following the different treatments. The results demonstrated that low-dose simvastatin had no evident affect on DU145 cell apoptosis (2.5±1.1%) compared with the control (1.7±0.8%) and LFU alone induced cell apoptosis (4.3±1.9%). The apoptosis rate significantly increased in the LFUM group (8.4±2.0%) and the LFU and simvastatin groups (15.8±2.2%). The combination of LFUM and simvastatin had a greater apoptosis rate of 33.9±4.6%. LFU marginally promoted cell apoptosis. Microbubbles or simvastatin increased the apoptosis rate of DU145 cells. LFUM combined with simvastatin was identified to induce an ~4-fold higher cell apoptotic rate than that of LFUM, and 2.2-fold greater than that of LFU and simvastatin. The difference was statistically significant (P<0.05). These results demonstrated that LFUM combined with simvastatin induced a strong synergistic effect on DU145 cell apoptosis (Fig. 3).

### LFUM combined with simvastatin decreases caveolin-1 expression

**Figure 4:**
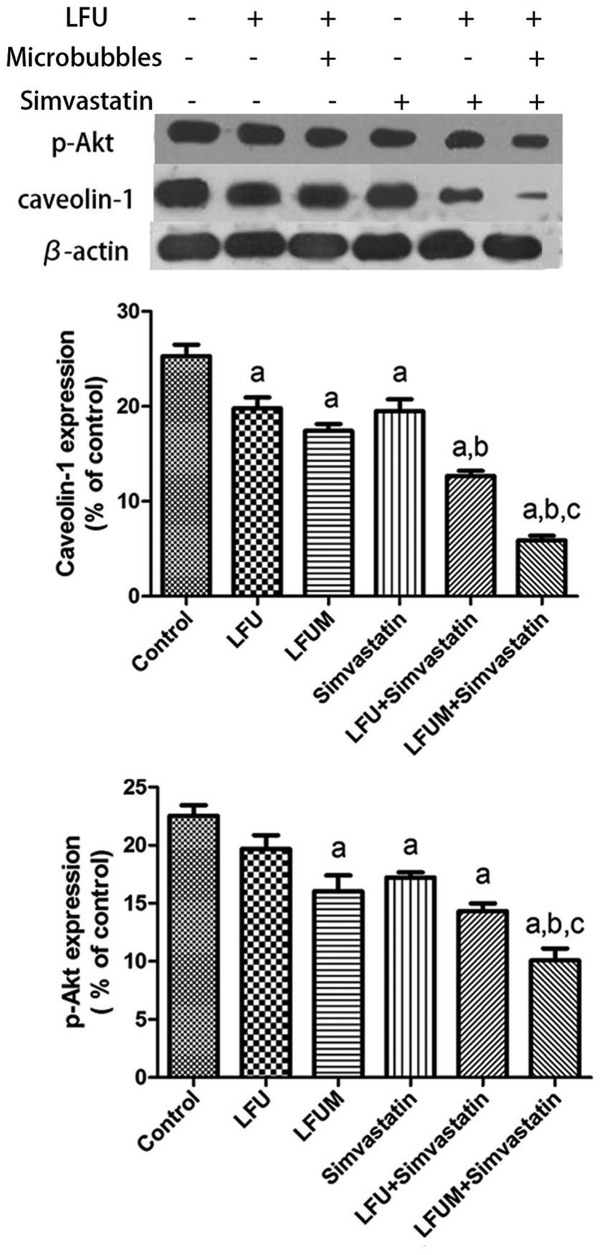
Inhibition of caveolin-1 and p-Akt production in human prostate cancer cells. DU145 cells were treated with the control, LFU, LFUM, simvastatin, LFU+simvastatin and LFUM+simvastatin. Western blotting was performed to examine the caveolin-1 and p-Akt expression for 24 h following ultrasound exposure. Error bars represent the mean ± standard deviation of three independent experiments. ^a^P<0.05 compared with the control; ^b^P<0.05 compared with the LFUM-treated cells; ^c^P<0.05 compared with the combined LFU and simvastatin-treated cells. LFU, low frequency ultrasound; LFUM, low frequency ultrasound and microbubbles; p-Akt, phospho-Akt.

As demonstrated by the western blot analysis, all of the five treatment groups exhibited a reduced expression of caveolin-1 in comparison with the control (25.3±2.1%). LFU inhibited the expression of caveolin-1 (19.8±2.0%) in a comparable manner to simvastatin (19.5±2.2%). The more effective treatments were LFUM (17.4±1.2%) and LFU combined with simvastatin (12.7±1.0%), which significantly reduced the level of caveolin-1 following 24 h of incubation. However, a more prominent decrease in the production of caveolin-1 occurred when the cells were treated with LFUM and simvastatin (5.9±0.8%). These results indicate that LFUM potentiates the inhibitory effect of simvastatin on the production of caveolin-1 (Fig. 4).

### Akt signaling is downregulated by LFUM combined with simvastatin

The anticancer mechanisms of simvastatin in prostate cancer cells has been associated with inhibition caveolin-1-dependent cell-survival signals, which are mediated via Akt activation (13,14). To investigate the affect of LFUM in combination with simvastatin on the production of p-Akt in DU145 cells, a western blot assay was performed. The DU145 cell line exhibited a decreased production of p-Akt following a 24 h exposure to LFU (19.7±2.1%) and simvastatin (17.2±0.8%) compared with the control (22.5±1.6%; Fig. 4). When the cells were subjected to combined LFU with simvastatin (14.3±1.1%) or LFUM (16.0±2.4%), the levels of p-Akt were lower than those produced by DU145 cells that were treated with LFU or simvastatin separately. The most potent inhibitory effect was observed in the culture that was treated with a combination of LFUM and simvastatin (10.1±1.8%), with an ~2.5-fold decrease in p-Akt production compared with the control. The decreases in caveolin-1 and p-Akt production were correlated. The data indicates that the combination of LFUM with simvastatin results in an additive effect in inhibiting the phosphorylation of Akt.

## Discussion

The biological impacts of LFUM on cancer cells are closely associated with ultrasound intensity, irradiation time and microbubble density. Cell death may occur with prolonged exposure or increased intensity. To induce cell apoptosis, rather than necrosis and death, the ultrasonic parameters were optimized in the pilot experiment. Ultrasound exposure induces tumor cell apoptosis (11,16,17) and this effect is enhanced by microbubbles, through the reduction of the cavitation threshold (12). In the present study, it was identified that low-frequency, low-intensity and short-exposure ultrasound with microbubbles promoted apoptosis of DU145 cells, and inhibited cell viability. However, the rate of apoptosis was very low and the antitumor effect was weak. Therefore, the additive/synergistic effect between LFUM and simvastatin on DU145 cells was subsequently observed.

Statin (simvastatin), the inhibitor of the mevalonate pathway, regulates cholesterol synthesis. Reliable evidence from *in vitro* and *in vivo* data has demonstrated that statins exert pleiotropic actions beyond their lipid-lowering effects, including in cancer prevention and treatment (6,18). Previous studies have also reported that statins trigger cancer cell apoptosis in various cancer cell types (19,20). The results from the present study revealed that short-time use of low-dose simvastatin had a very limited effect in inhibiting cell viability and inducing cell apoptosis at 24 h following treatment. Furthermore, we observed that sub-therapeutic simvastatin as well as LFU induced apoptosis and inhibited growth *in vitro* in prostate cancer cells, and the combination was more effective than either of them alone. Additionally, it was demonstrated that microbubbles enhanced the apoptosis of DU145 cells induced by a combination of LFU and simvastatin. LFUM combined with simvastatin exhibited the highest antitumor effect by inhibiting cell growth and inducing apoptosis on prostatic DU145 cells. The results indicate for the first time, to the best of our knowledge, an additive or a synergistic effect in so-called triple rescue regimens.

Caveolin-1 is a scaffolding protein and participates in regulating and concentrating specific lipids as well as modifying signaling molecules. Caveolin-1, through phosphorylation and/or dephosphorylation, interacts with signaling molecules, and regulates tumor cell proliferation, apoptosis, adhesion and movement (21). However, the function of caveolin-1 is cell- and tissue-specific. In ovarian (22), lung (23) and breast cancer tumors (24), the expression level of caveolin-1 is low, which leads to malignant growth when it is suppressed. Despite this, it is generally considered that overexpression of caveolin-1 is closely correlated with the occurrence of prostate cancer (4,25,26). In the present study, it was identified that the expression level of caveolin-1 was high in the resting DU145 cells. The level of caveolin-1 was lowered to a certain degree by treatment with either simvastatin or LFUM, and further decreased when they were applied in combination. In a previous study, statin (pravastatin) elicited a decrease of caveolin-1 expression in prostatic PC-3 cells (27). The inhibitory effect was explained by the reduced geranylgeranyl diphosphate level; specifically, the distribution of caveolin-1 was altered from the membrane to the cytoplasm during bisphosphonate treatment in PC-3 cells (27). Furthermore, simvastatin affected the lipid structure of the cell membrane through inhibition of the biosynthesis of cholesterol. Previous studies revealed pore-like structures in the cell membrane following treatment with ultrasound either with or without microbubbles (12,28). In addition to evoking transient pore formation, LFUM also triggered endocytosis, which was demonstrated by ion influx and cellular content release (29). As a result, the homeostasis balance of cells was destroyed, influencing the formation of a cell survival microenvironment. In addition, LFUM directly led to cell membrane destruction. When LFUM was combined with simvastatin, higher membrane permeability may have resulted due to decreased membrane integrity and stability, which prevented the formation of lipid rafts (27). Therefore, caveolin-1 may be released into the cytoplasm, leading to its degradation and decreased expression (12,28,29).

There are numerous membrane-bound proteins and cell signaling pathways in caveolae, including Akt and G protein-coupled receptors. Akt is a type of serine/threonine kinase that is significant in cell proliferation, apoptosis and angiogenesis. The direct association between caveolin-1 and Akt, which may be mediated through caveolin-1 binding to a caveolin-1 scaffolding domain-binding site on protein phosphotase (PP)1 and PP2A, and inhibition of their activities, results in significantly increased levels of p-Akt and sustained activation of downstream oncogenic Akt targets (14,26). Zundel *et al* (30) identified that caveolin-1 prevented cell apoptosis and promoted survival by activating the phosphatidylinositide 3-kinase (PI3-K)/Akt cell survival pathway. Several previous studies have also demonstrated that caveolin-1 stimulates angiogenic responses in prostate cancer cells through a mechanism that involves the PI3-K/Akt pathway (26,31). The anticancer efficacy of simvastatin for prostate cancer *in vitro* and *in vivo* has been associated with the inhibition of Akt expression (15). In the present study, LFUM combined with simvastatin reduced the level of caveolin-1, thereby inhibiting the activation of the downstream signaling molecule Akt (via phosphorylation), which was confirmed by western blot analysis. As a result, LFUM combined with simvastatin may act by disrupting the biosynthesis of cholesterol, decreasing the level of caveolin-1 and inhibiting p-Akt expression, leading to cell apoptosis and inhibition of cell viability. Therefore, LFUM combined with low-dose simvastatin may have important implications for chemoprevention and the treatment of prostate cancer.

In conclusion, the combination of LFU with microbubbles was demonstrated to enhance anti-prostate cancer activity. This effect was observed as enhanced growth inhibition, induction of apoptosis, and decreased caveolin-1 and p-Akt production. Furthermore, the additional application of sub-therapeutic doses of simvastatin resulted in enhanced apoptosis of DU145 cells concomitant with decreased caveolin-1 activation, as well as Akt phosphorylation. These results indicate that it may be possible to employ simvastatin together with a combination of LFU and microbubbles in refractory prostate cancer treatment.

## Figures and Tables

**Table I:** Results of cell apoptosis using orthogonal design analysis.

Number	Ultrasound intensity (W/cm^2^)	Time (sec)	Microbubble/cell suspension volume (%)	Apoptosis (%)
1	0.15	10	10	1.58
2	0.15	20	20	3.80
3	0.15	30	50	4.82
4	0.30	10	20	4.29
5	0.30	20	50	3.52
6	0.30	30	10	3.57
7	0.45	10	50	5.81
8	0.45	20	10	6.25
9	0.45	30	20	6.47
K_1_	10.20	11.68	11.40	
K_2_	11.38	13.57	14.56	
K_3_	18.53	14.86	14.15	
R	2.78	1.06	1.05	

K_1_, K_2_ and K_3_, the sum of three levels respectively; R, range.
